# Twenty-five-year trends in mortality and major morbidity among very low birth weight infants at a Saudi tertiary centre: improving morbidity despite expanding resuscitation at the limits of viability

**DOI:** 10.3389/fped.2026.1881490

**Published:** 2026-07-09

**Authors:** Lana A. Shaiba, Adnan Hadid, Khalid Almoosa, Abdulmalik Alharbi, Khalid Alharbi, Mohammed Albabtain, Badr Sobaih

**Affiliations:** 1 Department of Pediatrics, College of Medicine, King Saud University, Riyadh, Saudi Arabia; 2 Neonatology, King Saud University Medical City, Riyadh, Saudi Arabia

**Keywords:** neonatal morbidity, neonatal mortality, preterm infant, Saudi arabia, very low birth weight

## Abstract

**Background:**

Very low birth weight (VLBW) infants remain at high risk of mortality and major morbidity, yet longitudinal data from Saudi Arabia spanning several decades are scarce. We examined 25-year trends in survival and morbidity at a single tertiary centre, with particular attention to whether patterns reflect evolving case-mix at the limits of viability.

**Methods:**

This retrospective cohort study included inborn VLBW infants (birth weight <1,500 g, gestational age <33 weeks) admitted to the NICU of King Saud University Medical City, Riyadh, across three periods: 1999–2007 (*n* = 468), 2011–2018 (*n* = 512), and 2019–2024 (*n* = 396). Infants <22 weeks’ gestation or with lethal congenital anomalies were excluded. The primary outcome was survival to hospital discharge. Secondary outcomes were respiratory distress syndrome, bronchopulmonary dysplasia, patent ductus arteriosus, intraventricular haemorrhage, periventricular leukomalacia, necrotising enterocolitis, retinopathy of prematurity, and culture-proven sepsis. Categorical variables were compared using chi-square or Fisher's exact tests, continuous variables with ANOVA or Kruskal–Wallis tests. Multivariable regression was precluded by aggregate historical data; a sensitivity analysis restricted to infants ≥24 weeks examined case-mix effects.

**Results:**

Crude survival declined significantly across periods (88.7%, 84.0%, 79.0%; *p* = 0.001). However, among infants ≥24 weeks, survival was 90.0%, 85.8%, and 83.6%, indicating the overall decline is largely driven by increasingly admitted infants at 22–24 weeks. Survival at 23 weeks fell from 61.0% to 6.3%, while survival at 27–31 weeks remained above 80% without significant change. Morbidity improvements were substantial: early-onset sepsis fell from 11.0% to 2.3%, late-onset sepsis from 37.2% to 17.2%, necrotising enterocolitis from 15.6% to 9.3%, and severe retinopathy requiring treatment from 34.5% to 6.3% (all *p* < 0.001). Respiratory distress syndrome prevalence rose to 99.5%, while surfactant and postnatal corticosteroid use declined. Pneumothorax increased from 5.0% to 10.4% and periventricular leukomalacia from 1.3% to 6.6%; severe intraventricular haemorrhage remained unchanged.

**Conclusion:**

Expanding active resuscitation at 22–24 weeks generated an apparent decline in crude survival, while care quality markedly improved, evidenced by large reductions in sepsis, necrotising enterocolitis, and severe retinopathy. Caution is warranted in attributing the decline in crude survival solely to the change in resuscitation policy at 22–24 weeks, as evolving clinical management strategies and other policy changes over the study period may also have contributed to the observed outcomes. Standardised national benchmarking and multicentre surveillance are needed.

## 1 Introduction

Very low birth weight (VLBW) infants, defined as those with a birth weight below 1,500 g, are among the most vulnerable patients in neonatal medicine (1). Their physiological immaturity drives substantial risks of respiratory failure, sepsis, feeding intolerance, and thermoregulatory instability, and most require neonatal intensive care unit (NICU) admission (1, 2). Although VLBW infants represent only a small fraction of live births, they contribute disproportionately to neonatal mortality and to long-term neurodevelopmental burden worldwide (1).

Decades of progress in perinatal and neonatal care, including antenatal corticosteroids, exogenous surfactant, lung-protective ventilation, and evidence-based infection-prevention bundles, have improved survival of VLBW infants globally (3–5). Survival at the limits of viability, however, remains low, and survival without major morbidity continues to be the more demanding goal, particularly for the most immature infants (5, 6).

The global burden of prematurity is unevenly distributed. Data from the National Institute of Child Health and Human Development (NICHD) Neonatal Research Network document continued improvements in survival and morbidity among extremely preterm infants in the United States (7). In low- and middle-income settings, the picture is less favourable: a South African single-centre cohort reported a mortality rate of 32% among admitted VLBW neonates (8). In Saudi Arabia, several institutional studies have reported short-term VLBW outcomes (9–14), but only one previously addressed long-term temporal trends within a single institution (12), and few have systematically examined how case-mix at the limits of viability has shifted over time.

In Saudi Arabia, neonatal intensive care has expanded substantially over the past three decades, but practice at the limits of viability has historically been heterogeneous, and no single national threshold for active resuscitation has been uniformly applied. Earlier Saudi reports describe selective resuscitation at 22–25 weeks, with active care frequently withheld or withdrawn at the lowest gestations. At our centre, active resuscitation in the first two study periods (1999–2007 and 2011–2018) required both a gestational age of at least 22 completed weeks and a birth weight above 500 g. In the third period (2019–2024), this policy was changed so that all infants born at 22 weeks or more were offered active resuscitation regardless of birth weight, provided no lethal congenital anomaly was present. This local shift parallels the international move toward proactive care at 22–23 weeks that followed the 2015 revision of the Neonatal Resuscitation Program and subsequent guidance from several national bodies. The study period therefore spans a genuine change in who was offered resuscitation, which is central to the interpretation of our survival trends.

Regional evidence on resuscitation practice at the threshold of viability remains limited and is drawn almost entirely from single centres, with no published national or Gulf-wide consensus on a gestational-age threshold for active treatment. In Saudi Arabia, Ministry of Health guidance has historically favoured palliative care below 24 weeks, and a religious ruling permitting non-resuscitation before completion of the sixth lunar month has further shaped practice at the lowest gestations (15). The largest Saudi series to address this directly, from King Faisal Specialist Hospital and Research Centre (2006–2015), reviewed infants born at 23–25 weeks and reported that resuscitation decisions and survival were strongly gestation-dependent: at 23 weeks a substantial proportion received comfort care only or had active care withheld, and no infant survived free of major morbidity, whereas full support was initiated in most infants by 25 weeks (15). A separate report from central Saudi Arabia noted the absence of written national criteria for the lower limit of viability and called for such a standard, observing that birth weight may predict viability more reliably than gestational age in extremely low birth weight infants (11). Comparable Gulf data are sparse: a single-centre United Arab Emirates cohort (Tawam Hospital, 22–24 weeks) reported overall periviable survival of 18%, rising from 3% at 22 weeks to 46% at 24 weeks (16). To our knowledge, no multicentre Saudi or Gulf cohort has tracked how resuscitation thresholds and periviable outcomes have changed over time, which is the gap the present study addresses. Internationally, the trend toward proactive care at 22–23 weeks has been codified in frameworks such as the 2019 British Association of Perinatal Medicine guidance on management at extreme preterm gestations, against which our local change can be situated (17).

Tracking temporal trends is essential for benchmarking, identifying emerging problems, and evaluating quality-improvement initiatives (12). Accurate outcome data also support informed parental counselling, resource allocation, and the design of follow-up programmes for VLBW infants (13, 14, 18). Despite this, longitudinal Saudi data spanning several decades remain scarce. Two prior reports from our institution, King Saud University Medical City (KSUMC), described outcomes for 1999–2007 and 2011–2018 (13, 14). The present study extends this work with a third cohort (2019–2024) and compares outcomes across the full 25-year span, providing a unique longitudinal perspective from a single Saudi tertiary centre. Our objective was to evaluate trends in mortality and major morbidity among VLBW infants across the three periods and, given an evolving regional and institutional policy of active resuscitation at the limits of viability, to determine whether the apparent trends in survival are explained by case-mix shifts rather than by changes in the quality of care.

## 2 Materials and methods

### 2.1 Study design and setting

**Figure 1:**
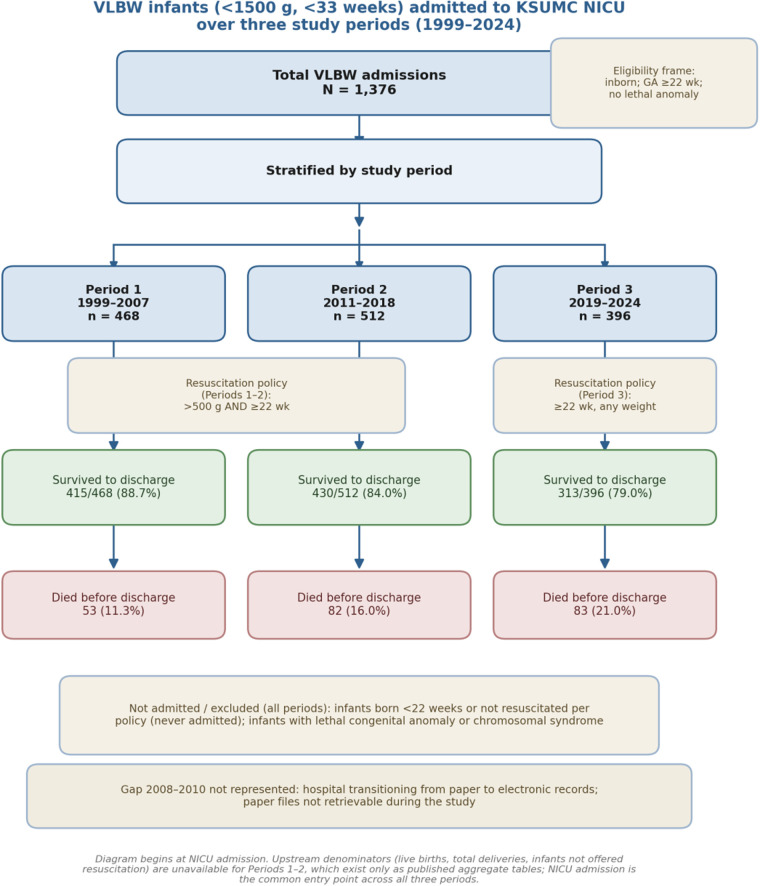
Flow of very low birth weight infants through the three study periods. The diagram begins at NICU admission, the common entry point across all three periods, because upstream denominators (live births, total deliveries, and infants not offered resuscitation) are not available for Periods 1 and 2, which exist only as published aggregate tables. Period definitions and the institutional resuscitation-threshold change at the Period 2/3 boundary (from “>500 g and ≥22 weeks” to “≥22 weeks regardless of weight”) are annotated, as is the 2008–2010 gap (paper-to-electronic record transition). Infants born below 22 weeks or not resuscitated per policy were never admitted, and infants with lethal congenital anomalies or chromosomal syndromes were excluded.

We conducted a retrospective analysis of routinely collected clinical data on VLBW infants admitted to the NICU at KSUMC, a university-affiliated tertiary care hospital in Riyadh, Saudi Arabia. The study compared three cohorts across distinct time periods: Period 1 (1999–2007), Period 2 (2011–2018), and Period 3 (2019–2024). Data for Periods 1 and 2 were re-extracted from the institutional registry where possible; where individual-level records were no longer accessible, aggregate summaries from prior peer-reviewed publications based on the same registry were used (13, 14). Period 3 data were drawn directly from the institutional registry. Reporting follows the STROBE recommendations for observational studies (checklist provided as Supplementary File 1). The interval 2008–2010 is not represented in this analysis. During those years the hospital was transitioning from paper-based to electronic medical record documentation, and the paper records for that period were not accessible during the study; comparable data could therefore not be retrieved. The flow of infants through the three periods is summarised in Figure 1.

### 2.2 Study population

All live-born infants with a birth weight <1,500 g and a gestational age <33 weeks admitted to the KSUMC NICU during the three periods were eligible. All infants were inborn. Infants born at less than 22 weeks gestation were not offered active resuscitation per institutional policy and were not included. In Period 1, active resuscitation was offered only to infants of ≥22 weeks gestation with a birth weight >500 g; resuscitation thresholds were progressively liberalised over Periods 2 and 3, with active resuscitation now offered from 22 completed weeks irrespective of birth weight when consistent with parental wishes. Infants with lethal congenital anomalies or chromosomal syndromes were excluded. The final sample comprised 1,376 infants (Period 1: 468; Period 2: 512; Period 3: 396).

### 2.3 Data collection

A standardised case-report form was used across all three periods to maximise comparability. Variables captured were maternal characteristics (age, parity); pregnancy and intrapartum factors (antenatal corticosteroid exposure, antenatal antibiotics, premature rupture of membranes); perinatal variables (mode of delivery, delivery-room resuscitation or intubation, Apgar scores at 1 and 5 min, admission temperature); neonatal demographics [sex, gestational age, birth weight, small-for-gestational-age (SGA) status]; and postnatal management (surfactant, postnatal corticosteroids, mechanical ventilation, length of stay). The data sources differed by period. Period 3 (2019–2024) data were collected retrospectively at patient level from the hospital electronic medical record and neonatal registry, then entered and cross-checked against the EMR by the study team using the standardised case-report form. Periods 1 (1999–2007) and 2 (2011–2018) were extracted from the aggregate tables of the two previously published KSUMC reports (13, 14); these are summary-level data. The earlier periods could not be re-abstracted at patient level because the original source records predate full electronic documentation and were not available, only the published aggregates.

### 2.4 Outcome definitions

The primary outcome was survival to hospital discharge. Secondary outcomes, with definitions held constant across periods, were: RDS, by clinical and radiographic features consistent with surfactant deficiency; BPD, supplemental-oxygen requirement at 36 weeks postmenstrual age; PDA, echocardiographically confirmed; IVH, graded according to Papile (19); PVL, diagnosed by cranial ultrasonography; NEC, modified Bell stage ≥ II (20); ROP, staged according to the International Classification (21); early-onset sepsis (EOS), culture-proven within the first 72 h; and late-onset sepsis (LOS), culture-proven after 72 h.

### 2.5 Ethics

This study was approved by the Institutional Review Board of King Saud University (reference E-25-9903). The requirement for informed consent was waived in view of the retrospective design and the use of de-identified data.

### 2.6 Statistical analysis

Analyses were performed in IBM SPSS Statistics version 25.0 (IBM Corp., Armonk, NY, USA). Categorical variables were summarised as frequencies and percentages, with chi-square or Fisher's exact tests for between-period comparisons (Monte Carlo simulation where >20% of expected cell counts were <5). Continuous variables were tested for normality using the Shapiro–Wilk test; normally distributed variables were compared by one-way ANOVA with Tukey *post-hoc* adjustment, and skewed variables (e.g., length of stay) by Kruskal–Wallis with Dunn's correction. A multivariable logistic regression for mortality could not be performed because individual-level data from Periods 1 and 2 were unavailable. To address case-mix shifts at the limits of viability, a pre-specified sensitivity analysis restricted to infants ≥24 weeks gestation was conducted, comparing survival across periods. This subgroup analysis was computed by summing the gestational-age strata for each period and is a crude, unadjusted comparison. In addition, a multivariable logistic regression for survival to discharge was fitted within Period 3, the only period with individual-level data, modelling survival against gestational age, birth weight, sex, small-for-gestational-age status, antenatal steroid exposure, and 5-min Apgar score. This adjusted analysis applies to the contemporary cohort only and could not be extended to Periods 1 and 2. Two-sided *p* < 0.05 was considered statistically significant.

## 3 Results

### 3.1 Baseline characteristics

A total of 1,376 VLBW infants were included (Period 1: 468; Period 2: 512; Period 3: 396). In Period 3, male infants comprised 50.6% (*n* = 201) and females 49.4% (*n* = 196). Most infants (97.7%) were admitted directly from labour and delivery, and 68.5% were delivered by emergency caesarean section. Delivery-room resuscitation or intubation was required in 53.7% of cases; 2.8% received resuscitation medications. An abnormal admission temperature was documented in 37.8% of infants. Antenatal antibiotics were given to 42.3% of mothers, premature rupture of membranes occurred in 29.0%, and 20.7% of infants were SGA (Table 1).

**Table 1:** Baseline maternal and neonatal characteristics, period 3 (2019–2024; *n* = 396).

Characteristic	n (%) or mean ± SD
Male sex	201 (50.6)
Female sex	196 (49.4)
Admitted from labour and delivery	387 (97.7)
Emergency caesarean delivery	271 (68.5)
Delivery-room resuscitation or intubation	213 (53.7)
Resuscitation medications	11 (2.8)
Abnormal admission temperature	150 (37.8)
Antenatal antibiotics	168 (42.3)
Premature rupture of membranes	115 (29.0)
Small for gestational age	82 (20.7)

Mean birth weight rose modestly from 993 ± 287 g in Period 1 to 1,038 ± 276 g in Period 2 and was 1,028.8 ± 295.1 g in Period 3 (*p* = 0.037), the increase being confined to the Period 1-to-2 transition with no further rise into Period 3. Maternal parity rose significantly across periods (*p* < 0.001). Apgar scores at both 1 and 5 min varied significantly: Period 3 values (5.20 ± 2.1 and 7.18 ± 1.7) were lower than those in Period 2 (5.7 ± 1.8 and 7.7 ± 1.4), and comparable with Period 1. Mean admission temperature improved progressively from 35.7 ± 1.0 °C to 37.1 ± 1.0 °C (*p* < 0.001) (Table 2). The standard deviation of birth weight in Period 3 is now comparable with the earlier periods following correction of a single data-entry error (a weight originally recorded as 7,820 g corrected to 782 g); the apparent further increase in mean birth weight into Period 3 in the earlier draft was an artefact of that outlier and is not real.

**Table 2:** Demographic characteristics across three periods (mean ± SD).

Variable	Period 1 (1999–2007) *n* = 468	Period 2 (2011–2018) *n* = 512	Period 3 (2019–2024) *n* = 396	p	Pairwise
Parity	1.0 ± 0.1	1.02 ± 0.17	1.7 ± 0.46	<0.001	*p*1 = 0.024; *p*2 < 0.001; *p*3 < 0.001
Gestational age (weeks)	27.7 ± 2.41	27.9 ± 2.42	27.85 ± 2.64	0.426	NS
Birth weight (g)	993 ± 287	1,038 ± 276	1,028.8 ± 295.1	0.037	*p*1 = 0.037; *p*2 = 0.157; *p*3 = 0.880
Apgar 1 min	5 ± 2	5.7 ± 1.8	5.20 ± 2.1	<0.001	*p*1 < 0.001; *p*2 = 0.144; *p*3 = 0.0002
Apgar 5 min	7.4 ± 1.5	7.7 ± 1.4	7.18 ± 1.7	<0.001	*p*1 = 0.001; *p*2 = 0.039; *p*3 < 0.001
Admission temperature ( °C)	35.7 ± 1.0	36.2 ± 0.4	37.1 ± 1.0	<0.001	*p*1 < 0.001; *p*2 < 0.001; *p*3 < 0.001
Antenatal steroids (any)	77.5%	81.5%	86.6%	0.003	—
Small for gestational age	12.5%	11.5%	20.7%	<0.001	—
Caesarean delivery	47.8%	58.6%	68.7%	<0.001	—
Antenatal antibiotics	33.8%	33.4%	41.7%	0.013	—
Premature rupture of membranes	3.4%	18.9%	29.0%	<0.001	—
Multiple birth	29.6%	29.3%	33.6%	0.327	NS

*p*1, period 1 vs. 2; *p*2, period 1 vs. 3; *p*3, period 2 vs. 3. *p* < 0.05 considered statistically significant.

### 3.2 Survival to discharge

Crude survival to discharge fell across the three periods: 88.7% (415/468), 84.0% (430/512), and 79.0% (313/396) refer to Table 3. Once analysis was restricted to infants born at ≥24 weeks gestation, survival was 90.0% in Period 1 (404/449), 85.8% in Period 2 (423/493), and 83.6% in Period 3 (312/373). The attenuation of the decline in this subgroup indicates that a substantial component of the apparent overall fall in survival is attributable to the rising proportion of admissions at 22–24 weeks, reflecting the evolution of the institutional resuscitation policy. Fifty-five infants were born at 22–24 weeks in Period 3 (13.9% of admissions); their characteristics and outcomes are detailed in Table 4. Survival in this group was 32.7% overall (0% at 22 weeks, 6.2% at 23 weeks, 53.1% at 24 weeks) against near-universal RDS and surfactant exposure and high rates of pneumothorax (27.3%) and BPD (36.4%) Table 4.

**Table 3:** Survival-to-discharge rates based on gestational age.

Gestational age (weeks)	KSUMC First period (1999–2007) (*N* = 468)		KSUMC Second period (2011–2018) (*N* = 512)		KSUMC Third period (2019–2024) (*N* = 396)		*p*-value^‡^
	Survived	Survival rate (%)	Survived	Survival rate (%)	Survived	Survival rate (%)	
22 weeks	0/1	0%	0/2	0%	0/7	0.0%	NA
23 weeks	11/18	61%	7/17	41.2%	1/16	6.3%	0.002
24 weeks	26/40	65%	15/37	40.5%	17/32	53.1%	0.080
25 weeks	28/33	84.8%	27/35	77.1%	18/25	72.0%	0.410
26 weeks	63/70	90%	44/60	73.3%	32/40	80.0%	0.040
27 weeks	44/49	90%	46/49	93.9%	36/44	81.8%	0.480
28 weeks	58/62	93.6%	85/94	90.4%	57/65	87.7%	0.730
29 weeks	54/59	91.5%	60/67	89.6%	46/53	86.8%	0.810
30 weeks	77/78	98.7%	76/80	95%	41/43	95.3%	0.750
31 weeks	39/43	91%	29/30	96.7%	40/43	93.0%	0.410
32 weeks	15/15	100%	41/41	100%	16/19	84.2%	0.040
33 weeks	NA	NA	NA	NA	7/7	100%	NA
34 weeks	NA	NA	NA	NA	2/2	100%	NA
Total	415/468	88.7%	430/512	84%	313/396	79%	0.001

*p* > 0.05 is non-significant; *p* ≤ 0.05 is significant *p* ≤ 0.05 is statistically significant. Data are presented as number (percentage).‏

‡ Chi-square test.

**Table 4:** Characteristics and outcomes of infants born at 22–24 weeks’ gestation, period 3 (2019–2024).

Characteristic	All 22–24 wk	*n*	22 wk	23 wk	24 wk	*p* (across wk)	Survivors n(%)	Non-surv n(%)	*p* (surv vs. not)
Total infants	56		8	16	32		19	37	
Survived to discharge	19 (33.9%)	19	1 (12.5%)	1 (6.3%)	17 (53.1%)		—	—	
Male	23 (41.1%)	23	5 (62.5%)	5 (31.3%)	13 (40.6%)	0.340^†^	10 (52.6%)	13 (35.1%)	0.208
Birth weight, mean (SD), g	624 (112)		516 (32)	556 (89)	685 (95)n	0.000	694	588	0.001
Antenatal steroid (any)	46 (82.1%)	46	4 (50.0%)	13 (81.3%)	29 (90.6%)	0.000^†^	18 (94.7%)	28 (75.7%)	0.078^†^
Resuscitation/intubation at birth	53 (94.6%)	53	7 (87.5%)	16 (100.0%)	30 (93.8%)	0.415^†^	16 (84.2%)	37 (100.0%)	0.013^†^
Resuscitation drugs	3 (5.4%)	3	0 (0.0%)	1 (6.3%)	2 (6.3%)	0.768^†^	0 (0.0%)	3 (8.1%)	0.202^†^
5-min Apgar <5	15 (26.8%)	15	2 (25.0%)	6 (37.5%)	7 (21.9%)	0.511^†^	2 (10.5%)	13 (35.1%)	0.049^†^
RDS	54 (96.4%)	54	7 (87.5%)	15 (93.8%)	32 (100.0%)	0.185^†^	18 (94.7%)	36 (97.3%)	0.625^†^
Surfactant (any)	53 (94.6%)	53	6 (75.0%)	15 (93.8%)	32 (100.0%)	0.019^†^	18 (94.7%)	35 (94.6%)	0.982^†^
Pneumothorax	15 (26.8%)	15	4 (50.0%)	3 (18.8%)	8 (25.0%)	0.249^†^	2 (10.5%)	13 (35.1%)	0.049^†^
BPD	20 (35.7%)	20	0 (0.0%)	4 (25.0%)	16 (50.0%)	0.018^†^	14 (73.7%)	6 (16.2%)	0.000
Severe IVH (grade 3–4)	6 (10.7%)	6	2 (25.0%)	1 (6.3%)	3 (9.4%)	0.350^†^	0 (0.0%)	6 (16.2%)	0.063^†^
PVL	4 (7.1%)	4	0 (0.0%)	2 (12.5%)	2 (6.3%)	0.510^†^	1 (5.3%)	3 (8.1%)	0.696^†^
NEC (≥ Bell II)	8 (14.3%)	8	0 (0.0%)	2 (12.5%)	6 (18.8%)	0.388^†^	3 (15.8%)	5 (13.5%)	0.818^†^
Any culture-proven sepsis	13 (23.2%)	13	0 (0.0%)	4 (25.0%)	9 (28.1%)	0.237^†^	7 (36.8%)	6 (16.2%)	0.083
ROP requiring treatment	8 (14.3%)	8	0 (0.0%)	0 (0.0%)	8 (25.0%)	0.030^†^	8 (42.1%)	0 (0.0%)	0.000^†^
Postnatal corticosteroids	19 (33.9%)	19	1 (12.5%)	6 (37.5%)	12 (37.5%)	0.384^†^	9 (47.4%)	10 (27.0%)	0.128

Denominators differ from 55 for variables with incomplete recording, shown as n/N. RDS, respiratory distress syndrome; BPD, bronchopulmonary dysplasia; IVH, intraventricular haemorrhage; PVL, periventricular leukomalacia; NEC, necrotising enterocolitis; ROP, retinopathy of prematurity.

*n* = 56 (GA 22–24 wks). Values are *n* (%) unless noted.

*p*-values: binary vars by chi-square (across-week = 2 df; survivor vs. non = 1 df); birth weight by one-way ANOVA (across week) and Welch t-test (survivor vs. non).

† Expected/observed cell count <5; chi-square approximate—interpret with caution (Fisher's exact preferred, not available in-cell). Survivor split: 19 survived vs. 37 died within 22–24 wk.

Stratification by gestational age was informative. No infant born at 22 weeks survived in any period. At 23 weeks, survival fell from 61.0% in Period 1 to 6.3% in Period 3 (*p* = 0.002). The relatively high survival at 23 weeks during Period 1 (61.0%, 11/18) should be interpreted in the context of the prevailing resuscitation threshold: during 1999–2007, only infants weighing more than 500 g and ≥22 weeks were offered active resuscitation. The denominator therefore reflects a highly selected cohort of 23-week infants, which likely explains the apparent discrepancy with contemporaneous NICHD figures. At 26 weeks, survival varied across periods (90.0%, 73.3%, and 80.0%; *p* = 0.040). Between 27 and 31 weeks, survival remained consistently above 80% with no significant temporal change. At 32 weeks, survival fell from 100% in Periods 1 and 2 to 84.2% in Period 3 (*p* = 0.040), although this is based on only 19 infants in Period 3 and warrants caution. The 3 deaths among these 19 infants had no common mechanism (two small-for-gestational-age, one with pneumothorax), and the finding is most consistent with small-number variation compounded by the higher SGA fraction in Period 3 rather than a deterioration in care. Gestational-age-specific survival is shown in Table 2.

#### 3.2.1 Multivariable analysis of survival (period 3)

A multivariable logistic regression for survival to discharge was fitted within Period 3, the only period with individual-level data (396 infants with complete covariates; 313 survivors, 83 deaths). After adjustment, higher gestational age [adjusted odds ratio (aOR): 1.36 per week, 95% CI: 1.12–1.66; *p* = 0.002], higher 5-min Apgar score (aOR: 1.26 per point, 95% CI: 1.07–1.48; *p* = 0.005), and any antenatal corticosteroid exposure (aOR: 2.28, 95% CI: 1.07–4.88; *p* = 0.033) were each independently associated with survival. Birth weight (aOR: 1.11 per 100 g, 95% CI: 0.93–1.33; *p* = 0.246), male sex (aOR: 1.35, 95% CI: 0.76–2.41; *p* = 0.304), and small-for-gestational-age status (aOR 0.66, 95% CI 0.30–1.43; *p* = 0.288) were not independently associated with survival once gestational age was accounted for (model McFadden pseudo-*R*^2^ 0.23; likelihood-ratio *p* < 0.001). Gestational age therefore dominated the contemporary survival signal, consistent with the case-mix interpretation. This adjusted analysis is confined to Period 3 and cannot be extended to Periods 1 and 2, which exist only as published aggregate tables (Table 5).

**Table 5:** Multivariable logistic regression for survival to discharge, period 3 (2019–2024; *n* = 396).

Variable	aOR (95% CI)	*p*-value
Gestational age (per week)	1.36 (1.12–1.66)	0.002
Birth weight (per 100 g)	1.11 (0.93–1.33)	0.246
Male sex	1.35 (0.76–2.41)	0.304
Small for gestational age	0.66 (0.30–1.43)	0.288
Antenatal corticosteroids (any)	2.28 (1.07–4.88)	0.033
5-min Apgar (per point)	1.26 (1.07–1.48)	0.005

aOR, adjusted odds ratio for survival to discharge; CI, confidence interval.

Outcome modelled was survival (vs. death). Model fitted on 396 Period 3 infants with complete covariates; McFadden pseudo-*R*^2^ = 0.23, likelihood-ratio *p* < 0.001. Cross-period modelling was not possible because Periods 1 and 2 are available only as published aggregates.

### 3.3 Respiratory outcomes

RDS prevalence rose to 99.5% in Period 3 (*p* < 0.001) while surfactant use fell from 80.0% in Period 1 to 63.4% in Period 3 (*p* < 0.001), consistent with the adoption of less-invasive respiratory support. Postnatal corticosteroid use decreased markedly from 27.9% to 11.6% (*p* < 0.001). Pneumothorax rose from 5.0% to 10.4% (*p* = 0.006). Overall BPD prevalence did not change significantly (27.4%, 23.6%, 26.8%; *p* = 0.361). Detailed morbidity data are presented in Table 6.

**Table 6:** Short-term outcomes and morbidities across three periods.

Outcome	Period 1 (*n* = 468)	Period 2 (*n* = 512)	Period 3 (*n* = 396)	*p*	Pairwise
RDS	446 (95.2)	453 (88.5)	394 (99.5)	<0.001	*p*1 < 0.001; *p*2 < 0.001; *p*3 < 0.001
Surfactant use	374 (80.0)	341 (66.6)	251 (63.4)	<0.001	*p*1 < 0.001; *p*2 < 0.001; *p*3 = 0.348
Pneumothorax	23 (5.0)	33 (6.5)	41 (10.4)	0.006	*p*1 = 0.372; *p*2 = 0.004; *p*3 = 0.044
BPD	128 (27.4)	121 (23.6)	106 (26.8)	0.361	NS
NEC ≥ Bell II	73 (15.6)	82 (16.0)	37 (9.3)	0.007	*p*1 = 0.927; *p*2 = 0.008; *p*3 = 0.004
PDA incidence	145 (31.0)	143 (27.9)	149 (37.6)	0.007	*p*1 = 0.328; *p*2 = 0.048; *p*3 = 0.002
PDA, medical Tx	70 (48.3)	24 (16.6)	81 (54.7)	<0.001	*p*1 < 0.001; *p*2 < 0.001; *p*3 = 0.161
PDA, surgical Tx	18 (12.4)	3 (2.2)	3 (2.0)	<0.001	*p*1 < 0.001; *p*2 < 0.001; *p*3 = 0.157
IVH grade 1	13 (2.7)	19 (3.7)	21 (5.3)	0.154	NS
IVH grade 2	16 (3.4)	13 (2.5)	24 (6.1)	0.020	*p*1 = 0.533; *p*2 = 0.093; *p*3 = 0.013
IVH grade 3	21 (4.4)	18 (3.5)	13 (3.3)	0.603	NS
IVH grade 4	16 (3.4)	18 (3.5)	11 (2.8)	0.805	NS
PVL	6 (1.3)	13 (2.5)	26 (6.6)	<0.001	*p*1 = 0.233; *p*2 < 0.001; *p*3 = 0.005
ROP (all stages)	161 (34.5)	145 (28.3)	126 (31.8)	0.120	NS
EOS	51 (11.0)	8 (1.6)	9 (2.3)	<0.001	*p*1 < 0.001; *p*2 < 0.001; *p*3 = 0.582
LOS	174 (37.2)	123 (24.0)	68 (17.2)	<0.001	*p*1 < 0.001; *p*2 < 0.001; *p*3 = 0.015
Postnatal steroids	131 (27.9)	41 (8.0)	46 (11.6)	<0.001	*p*1 < 0.001; *p*2 < 0.001; *p*3 = 0.086
Discharge weight, g (mean ± SD)	2,357 ± 740	2,507 ± 1,066	2,324 ± 912	0.006	*p*1 = 0.010; *p*2 = 0.539; *p*3 = 0.007
Survived	415 (88.7)	430 (84.0)	313 (79.0)	0.001	*p*1 = 0.042; *p*2 < 0.001; *p*3 = 0.067

RDS, respiratory distress syndrome; BPD, bronchopulmonary dysplasia; NEC, necrotising enterocolitis; PDA, patent ductus arteriosus; IVH, intraventricular haemorrhage; PVL, periventricular leukomalacia; ROP, retinopathy of prematurity; EOS, early-onset sepsis; LOS, late-onset sepsis.

Values are *n* (%) unless stated otherwise. *p*1 = Period 1 vs. 2; *p*2 = Period 1 vs. 3; *p*3 = Period 2 vs. 3.

### 3.4 Neurological outcomes

Severe IVH (grades 3–4) was stable across periods. Grade 2 IVH rose from 3.4% in Period 1 to 6.1% in Period 3 (*p* = 0.020), and PVL increased from 1.3% to 6.6% (*p* < 0.001). Grade 1 IVH did not differ significantly (2.7%, 3.7%, 5.3%; *p* = 0.154).

### 3.5 Infection and gastrointestinal outcomes

EOS fell from 11.0% in Period 1 to 2.3% in Period 3 (*p* < 0.001). LOS fell from 37.2% to 17.2% (*p* < 0.001). NEC ≥Bell stage II decreased from 15.6% to 9.3% (*p* = 0.007).

### 3.6 Ophthalmic and cardiovascular outcomes

Overall ROP across all stages did not differ significantly between periods (34.5%, 28.3%, 31.8%; *p* = 0.120). The rate of ROP requiring treatment fell from 34.5% in Period 1 to 6.3% in Period 3 (*p* < 0.001). PDA incidence rose modestly (31.0%, 27.9%, 37.6%; *p* = 0.007), but the management mix shifted substantially: medical treatment fell from 48.3% to 54.7% of PDA cases (*p* < 0.001) and surgical ligation from 12.4% to 2.0% of PDA cases (*p* < 0.001). All PDA treatment figures are expressed as a proportion of PDA cases in that period.

### 3.7 Discharge outcomes

Mean weight at discharge differed across periods (2,357 ± 740 g; 2,507 ± 1,066 g; 2,324 ± 912 g; *p* = 0.006), with Period 2 showing the highest mean discharge weight.

## 4 Discussion

This 25-year, three-period analysis of VLBW infants at a single Saudi tertiary centre produced a deliberately mixed picture. We observed substantial improvements in several key morbidities, including sepsis, NEC, severe ROP, and surgical PDA ligation, alongside an apparent decline in crude survival to discharge. These findings are not contradictory. Together they reflect a unit that has continued to improve preterm care while concurrently extending active resuscitation to the most immature gestational ages, where mortality remains very high.

### 4.1 Survival in the context of expanding resuscitation at the limits of viability

Crude survival fell from 88.7% in 1999–2007 to 79.0% in 2019–2024, but this pattern reflects an evolving denominator more than deteriorating care. The proportion of admissions at 22–24 weeks increased across periods, and survival at 23 weeks fell from 61.0% to 6.3%, in line with the explicit liberalisation of the institutional resuscitation policy. In Period 1, only infants of ≥22 weeks and >500 g were offered resuscitation, producing a highly selected denominator; by Period 3, active resuscitation was offered from 22 completed weeks when consistent with parental wishes, with a corresponding rise in admissions of infants at the lowest expected survival. The sensitivity analysis restricted to infants ≥24 weeks supports this interpretation: survival in this subgroup remained 84%–90% (90.0%, 85.8%, 83.6%) across all three periods and showed only modest attenuation. Where the denominator excludes the most immature infants, the apparent decline in survival largely disappears. Similar denominator effects have shaped reported trends in other long-running NICU cohorts (5, 7).

A multivariable logistic regression adjusting for gestational age, birth weight, sex, antenatal steroids, and SGA would be the strongest statistical demonstration of this case-mix effect. Such a model could not be fitted across all three periods in the present study because individual-level records from Periods 1 and 2 were no longer accessible and only aggregate published tables remained. Within Period 3, where individual-level data exist, the adjusted model (Table 5) showed that gestational age, 5-min Apgar, and antenatal steroid exposure independently predicted survival, while birth weight, sex, and SGA did not once gestational age was accounted for; this supports the case-mix interpretation for the contemporary cohort but cannot be extended backward. We have flagged this as a key limitation, and prospective multicentre work in a Saudi Neonatal Network would address it directly.

### 4.2 Changes in respiratory care and unintended consequences

Surfactant use fell from 80.0% to 63.4%, consistent with the shift toward early CPAP and selective rescue surfactant supported by the European Consensus Guidelines (22). The simultaneous rise in pneumothorax from 5.0% to 10.4% (*p* = 0.006) is a concern that should prompt quality-improvement action. Plausible contributors include delayed surfactant administration when CPAP fails, suboptimal CPAP pressure management in the most immature infants, and uneven operator experience during transition years. Comparable tensions between non-invasive support and air-leak risk have been described in NICHD data (7). Overall BPD prevalence was unchanged. In the context of rising survival pressure on the most immature infants, a stable BPD rate is best interpreted as a relative success; granular analyses by gestational age and BPD severity would refine that picture.

### 4.3 Sustained improvements in infection, NEC, and ROP

EOS fell from 11.0% to 2.3% and LOS from 37.2% to 17.2%. Reductions of this magnitude are consistent with the introduction of central-line bundles, hand-hygiene programmes, and antimicrobial-stewardship initiatives during the study period (7). NEC fell from 15.6% to 9.3%, in keeping with global evidence supporting standardised feeding protocols and early human-milk use (23). The fall in ROP requiring treatment from 34.5% to 6.3% almost certainly reflects more rigorous oxygen-saturation targeting; the magnitude exceeds that reported in many international cohorts and represents one of the most striking findings of this study. Surgical PDA ligation fell from 12.4% to 2.0% of PDA cases, mirroring the shift toward expectant management supported by the BeNeDuctus randomised trial (24).

### 4.4 Neurological outcomes and the role of surveillance

Severe IVH was unchanged across periods. Stable rates of grade 3–4 IVH against a background of expanded admission at 22–24 weeks should be considered a positive signal. The rises in grade 2 IVH and PVL most likely reflect more frequent and higher-resolution cranial ultrasonography rather than a deterioration in brain injury. Improving antenatal corticosteroid coverage over the period would be expected to protect against severe IVH (25). Where available, the number of cranial ultrasound studies per infant per period would directly support an ascertainment-bias interpretation, and we recommend that this be captured prospectively in future Saudi Neonatal Network reporting.

### 4.5 Implications for benchmarking and policy in Saudi Arabia

These data have practical implications. First, regional reporting of VLBW outcomes should always present gestational-age-stratified survival; aggregate survival figures are misleading when resuscitation thresholds shift. Second, the morbidity gains we observed indicate that targeted bundles (infection prevention, oxygen-saturation targeting, feeding protocols) are feasible and effective in our setting. Third, a national Saudi Neonatal Network would enable adjusted, multicentre benchmarking and would resolve questions that no single-centre dataset, however long, can fully answer.

#### 4.5.1 Evolution of unit practice across the three periods

Several documented changes in unit practice frame these trends. The central change is in resuscitation policy: the threshold moved at the start of Period 3 from “>500 g and ≥22 weeks” to “≥22 weeks regardless of weight,” which ties directly to the rise in 22–24-week admissions. Respiratory management shifted toward non-invasive-first support: surfactant use fell across periods (80.0% to 66.6% to 63.4%, *p* < 0.001) while RDS documentation rose, consistent with early CPAP and selective rescue surfactant in line with the 2022 European RDS guidelines (22); the concurrent rise in pneumothorax (5.0% to 10.4%, *p* = 0.006) is a plausible unintended consequence and a quality-improvement target. PDA management moved toward conservative care, with surgical ligation falling to 2.0% of PDA cases and medical treatment changing in parallel, supported by the BeNeDuctus trial (24). The falls in early- and late-onset sepsis are consistent with central-line and hand-hygiene bundles and antimicrobial stewardship, and mean admission temperature rose from 35.7 to 37.1 °C (*p* < 0.001), consistent with improved delivery-room thermal care. We are deliberately cautious with attribution: surfactant route (less-invasive surfactant administration) and delayed cord clamping are not recorded as discrete fields and cannot be dated or quantified from these data, so we discuss them as likely contributors in line with the secular trend rather than as measured changes.

#### 4.5.2 Unit policy for infants at 22–24 weeks

The policy for the most immature infants shifted decisively over the study. In Periods 1 and 2, active resuscitation required both ≥22 weeks and a birth weight >500 g, so a 22–24-week infant below that weight was not resuscitated, never reached the NICU, and never entered the denominator. From 2019 (Period 3), all infants ≥22 weeks were offered resuscitation regardless of weight, provided no lethal anomaly was present; every admitted infant was by definition actively resuscitated, with no separate comfort-care admission pathway in the dataset. Among the 55 Period 3 infants born at 22–24 weeks, 96.4% received delivery-room resuscitation or intubation and 32.7% survived (0% at 22 weeks, 6.2% at 23 weeks, 53.1% at 24 weeks). This rise in 22–24-week admissions in Period 3, and not before, coincides exactly with the local policy change at the Period 2/3 boundary, and parallels the international move toward proactive care at 22–23 weeks after the 2015 NRP revision and subsequent national frameworks. These infants are the high-acuity group whose growing numbers pull crude survival down while gestational-age-specific survival at 27–31 weeks stays excellent.

#### 4.5.3 The 32-week survival signal

Survival at 32 weeks fell from 100% in Periods 1 and 2 to 84.2% in Period 3, but this rests on only 19 infants with 3 deaths. The three deaths had no common mechanism: two were small-for-gestational-age (birth weights 800 g and 1,220 g, one with NEC) and one had a pneumothorax (1,290 g). With a denominator of 19, the confidence interval around 84.2% is wide and spans the earlier periods’ rates. This finding is most consistent with small-number variation, compounded by the higher SGA fraction in Period 3, rather than a deterioration in 32-week care, and should not be over-interpreted, though it warrants ongoing surveillance.

### 4.6 Strengths and limitations

The principal strength of this study is its 25-year span across three carefully defined periods at one Saudi tertiary centre, with consistent case definitions. Pairwise period comparisons add granularity. Limitations include the retrospective design, reliance on published aggregate data for Periods 1 and 2 (which precluded individual-level multivariable adjustment), absence of long-term neurodevelopmental follow-up, gap years (2008–2010) not captured, potential residual confounding from secular changes in obstetric practice, referral patterns, and unit staffing, and single-centre origin limiting generalisability. Several confounders beyond the resuscitation policy could contribute to the crude mortality trend and cannot be adjusted for given the aggregate earlier data: secular changes in obstetric referral patterns, with a tertiary centre potentially receiving sicker maternal-fetal transfers over time; the near-doubling of the small-for-gestational-age proportion (12.5% to 20.7%); the lower 5-min Apgar in Period 3; unit occupancy and staffing pressures; and the absence of long-term neurodevelopmental follow-up that would distinguish survival from intact survival. These represent residual confounding that the case-mix interpretation contextualises rather than eliminates, and that a prospective adjusted cohort would address. Because individual-level data exist only for Period 3, the cross-period case-mix interpretation rests on the stratified (gestational-age-specific and ≥24-week) analyses rather than on adjusted modelling.

## 5 Conclusion

Across 25 years at a single Saudi tertiary centre, we observed substantial reductions in sepsis, NEC, severe ROP, and surgical PDA ligation, alongside an apparent fall in crude overall survival. The survival pattern is best understood in the context of an expanding policy of active resuscitation at 22–24 weeks and the resulting case-mix shift toward higher-acuity admissions; gestational-age-specific survival between 27 and 31 weeks remained excellent and unchanged. The sensitivity analysis restricted to infants ≥24 weeks confirmed an attenuated survival decline, supporting a case-mix explanation. Standardised quality-improvement protocols, enhanced perinatal care, and national-level benchmarking through a Saudi Neonatal Network are needed to confirm and extend these findings.

## Data Availability

The raw data supporting the conclusions of this article will be made available by the authors, without undue reservation.
