# Supplementary material for: Twenty-five-year trends in mortality and major morbidity among very low birth weight infants at a Saudi tertiary centre: improving morbidity despite expanding resuscitation at the limits of viability
Source: Front Pediatr. 2026 Jul 9;14:1881490. doi: 10.3389/fped.2026.1881490 (PMC13391831; doi:10.3389/fped.2026.1881490)
Supplement: Supplementary file 1 [file Table1.docx]

**SUPPLEMENTARY FILE 1: STROBE Checklist for Cohort Studies**

The following checklist is based on the STROBE Statement — Checklist of items that should be included in reports of *cohort* studies (von Elm E, et al. The Strengthening the Reporting of Observational Studies in Epidemiology [STROBE] Statement. Ann Intern Med. 2007;147:573–577).

| **Item** | **No.** | **Recommendation** | **Reported on page / section** |
| --- | --- | --- | --- |
| Title and abstract | 1 | Indicate the study design with a commonly used term in the title or the abstract — and provide an informative, balanced summary of what was done and what was found. | Title page; Abstract |
| Background/rationale | 2 | Explain the scientific background and rationale for the investigation being reported. | Introduction |
| Objectives | 3 | State specific objectives, including any pre-specified hypotheses. | Introduction (final paragraph) |
| Study design | 4 | Present key elements of study design early in the paper. | Methods: Study Design |
| Setting | 5 | Describe the setting, locations, and relevant dates (recruitment, exposure, follow-up, data collection). | Methods: Study Design |
| Participants | 6a | Give eligibility criteria and the sources and methods of selection. | Methods: Study Population |
|  | 6b | Describe methods of follow-up. | Methods: Study Population |
| Variables | 7 | Clearly define all outcomes, exposures, predictors, potential confounders, and effect modifiers. | Methods: Outcome Definitions |
| Data sources / measurement | 8 | For each variable, describe data sources and methods of assessment. | Methods: Data Collection |
| Bias | 9 | Describe any efforts to address potential sources of bias. | Methods; Discussion: Limitations |
| Study size | 10 | Explain how the study size was arrived at. | Methods: Study Population |
| Quantitative variables | 11 | Explain how quantitative variables were handled. | Methods: Statistical Analysis |
| Statistical methods | 12a | Describe all statistical methods, including those used to control for confounding. | Methods: Statistical Analysis |
|  | 12b | Describe any methods used to examine subgroups and interactions. | Methods: Statistical Analysis |
|  | 12c | Explain how missing data were addressed. | Methods (N/A complete case analysis) |
|  | 12d | If applicable, describe analytical methods accounting for sampling strategy. | N/A |
|  | 12e | Describe any sensitivity analyses. | Methods: Statistical Analysis |
| Participants | 13 | Report numbers in/out at each stage of study with a flow diagram if possible. | Results: Baseline Characteristics |
| Descriptive data | 14 | Give characteristics of study participants; indicate number with missing data. | Results: Tables 1-2 |
| Outcome data | 15 | Report numbers of outcome events or summary measures. | Results: Tables 3-4 |
| Main results | 16 | Give unadjusted estimates and, if applicable, confounder-adjusted estimates. | Results |
| Other analyses | 17 | Report other analyses done (subgroup, sensitivity). | Results: Survival by GA; Discussion |
| Key results | 18 | Summarise key results with reference to study objectives. | Discussion |
| Limitations | 19 | Discuss limitations, considering sources of bias or imprecision. | Discussion: Strengths and Limitations |
| Interpretation | 20 | Give a cautious overall interpretation considering objectives, limitations, and other evidence. | Discussion; Conclusion |
| Generalisability | 21 | Discuss the generalisability of the study results. | Discussion: Strengths and Limitations |
| Funding | 22 | Give funding sources and role of the funders. | Declarations: Funding |
